# scMultiome analysis identifies a single caudal hindbrain compartment in the developing zebrafish nervous system

**DOI:** 10.1186/s13064-024-00189-z

**Published:** 2024-07-05

**Authors:** Jessica Warns, Yong-II Kim, Rebecca O’Rourke, Charles G. Sagerström

**Affiliations:** 1 Section of Developmental Biology, Department of Pediatrics, University of Colorado Medical School, 12801 E. 17th Avenue, Aurora, CO 80045 USA; 2 Department of Science and Math, Northern State University, 1200 S. Jay St, Aberdeen, SD 57401 USA

**Keywords:** Neuromere, Hindbrain, Spinal cord, Rhombomere, Chromatin state, Transcriptional regulation, Neural progenitor

## Abstract

**Background:**

A key step in nervous system development involves the coordinated control of neural progenitor specification and positioning. A long-standing model for the vertebrate CNS postulates that transient anatomical compartments – known as neuromeres – function to position neural progenitors along the embryonic anteroposterior neuraxis. Such neuromeres are apparent in the embryonic hindbrain – that contains six rhombomeres with morphologically apparent boundaries – but other neuromeres lack clear morphological boundaries and have instead been defined by different criteria, such as differences in gene expression patterns and the outcomes of transplantation experiments. Accordingly, the caudal hindbrain (CHB) posterior to rhombomere (r) 6 has been variably proposed to contain from two to five ‘pseudo-rhombomeres’, but the lack of comprehensive molecular data has precluded a detailed definition of such structures.

**Methods:**

We used single-cell Multiome analysis, which allows simultaneous characterization of gene expression and chromatin state of individual cell nuclei, to identify and characterize CHB progenitors in the developing zebrafish CNS.

**Results:**

We identified CHB progenitors as a transcriptionally distinct population, that also possesses a unique profile of accessible transcription factor binding motifs, relative to both r6 and the spinal cord. This CHB population can be subdivided along its dorsoventral axis based on molecular characteristics, but we do not find any molecular evidence that it contains multiple pseudo-rhombomeres. We further observe that the CHB is closely related to r6 at the earliest embryonic stages, but becomes more divergent over time, and that it is defined by a unique gene regulatory network.

**Conclusions:**

We conclude that the early CHB represents a single neuromere compartment that cannot be molecularly subdivided into pseudo-rhombomeres and that it may share an embryonic origin with r6.

**Supplementary Information:**

The online version contains supplementary material available at 10.1186/s13064-024-00189-z.

## Background

Formation of a functional central nervous system requires the generation of distinct neural progenitor cells at precisely defined locations during embryonic development. Disruptions to this process may lead to inappropriate neural circuit formation – such as an imbalance between excitatory and inhibitory circuits – and is thought to contribute to various neurodevelopmental disorders (e.g., autism spectrum disorder; [1, 2]). A key goal of developmental neurobiology is therefore to understand how neural progenitor specification is controlled in space and time during embryogenesis. In vertebrates, one model for this process postulates that the early neural primordium is transiently subdivided into repeated transverse compartments (neuromeres) along its anteroposterior (AP) axis, and that each neuromere supports the formation of a distinct set of neural progenitors [3]. Neuromeres are particularly prominent in the embryonic hindbrain, where they are named rhombomeres [4]. The rhombomeres are visually apparent transiently during embryogenesis and their existence is supported by distinct gene expression patterns in each rhombomere, as well as by cell proliferation differences and by the formation of unique neuron types within each rhombomere [5, 6].

The rostral limit of the embryonic hindbrain is clearly defined by a prominent constriction of the neural tube (known as the midbrain-hindbrain boundary) between the most rostral rhombomere (r1) and the adjacent midbrain. Similarly, the borders delineating r1 through r6 are morphologically distinct, but the arrangement of the hindbrain caudal to r6, and the nature of the boundary between the hindbrain and the spinal cord is less well defined. There are indications that the region of the embryonic hindbrain located caudal to r6 – usually referred to as the caudal hindbrain (CHB) – can be subdivided into additional rhombomere units. In mouse and chick, an additional morphological boundary is thought to exist in the CHB to separate two additional rhombomeres (r7 and r8), but r8 is not thought to be separated from the rostral spinal cord by an equivalent boundary [5, 7]. Some molecular support for the existence of r7 and r8 stem from observed differences in paralog group (PG) 4 *hox* gene expression levels within the caudal hindbrain that may coincide with the r7/r8 boundary [8]. More recently, closer parsing of *hox* gene expression patterns in embryonic chick and murine caudal hindbrain [9, 10], together with comparisons to neuroanatomical features and somitic boundaries, as well as chick transplantation experiments [11], have led to the proposal that the CHB consists of five distinct rhombomeres (r7-r11). Since these rhombomeres are not defined by visually apparent morphological boundaries, they have been termed pseudo- or crypto-rhombomeres [9–11]. If such pseudo-rhombomeres produce distinct neural derivatives, each would be expected to display a unique gene regulatory network (GRN), but it remains unclear if pseudo-rhombomeres can be defined by molecular differences beyond their *hox* gene expression profiles. Addressing this question has been challenging due to a lack of comprehensive molecular data for this region of the developing CNS. Indeed, initial scRNAseq analyses of the developing hindbrain were unable to fully resolve the developing rhombomeres [12–16]. We recently overcame this obstacle using scMultiome analysis (i.e., combined scATACseq and scRNAseq) in zebrafish [17]. In this manner, we were able to resolve r1-r6, to delineate GRNs for each of these rhombomeres, and to begin understanding their formation during embryogenesis. This analysis also detected progenitors that we assigned to the CHB and the rostral spinal cord (SC), but that have not yet been analyzed in detail. Here we use this scMultiome resource to identify a population of CHB cells that is distinct from both r6 and the SC at all stages analyzed and that possesses a unique GRN. This CHB population can be further subdivided along its dorsoventral axis, but we do not find any evidence that it can be molecularly subdivided into multiple rhombomeres or pseudo-rhombomeres. At the earliest stage analyzed, we also observe that the CHB shares more molecular features with r6 than with the spinal cord, suggesting that it may share a closer embryonic origin with r6. We conclude that the zebrafish hindbrain caudal to r6 cannot be molecularly subdivided into (pseudo)-rhombomeres at stages when r1-r6 are well-established, but we cannot exclude the possibility that such caudal rhombomeres form at later stages of embryogenesis or that they are present in other species.

## Methods

### Animals

The Institutional Animal Care and Use Committee of the University of Colorado Medical School approved all procedures involving zebrafish. Wildtype AB (ZL1) and TU (ZL57) zebrafish were obtained from the Zebrafish International Resource Center and reared in our facility. Embryos were collected in in egg water (60 µg/mL Instant Ocean, 0.0002% methylene blue), and maintained in an incubator at 29 °C. Dead and unfertilized eggs were manually removed prior to experimentation and were excluded from analyses.

### scMultiome sample preparation

The scMultiome data analyzed in this paper was generated previously (GEO record number GSE223535; [17]) and the sample preparation is briefly summarized here. Tissue containing the hindbrain was obtained from 25 *TG(hoxb1a: eGFP*^*um8*^*)* transgenic embryos (that express GFP in r4; [18]) at 13hpf and 16hpf by dissecting out the region between the eye and somite 5. At 10hpf, 100 whole embryos were pooled. Dead and unfertilized embryos were excluded from sample preparation. No other animals were excluded, and sample size was selected to achieve sufficient material for analysis and to smooth small variations in developmental stage. Tissue was collected in 1XPBS and dissociated by pipetting through a p1000 tip, followed by incubation in 500 µl of protease solution (10 mg/ml BI protease (Sigma, P5380), 125 U/ml DNase, 2.5 mM EDTA in PBS) for 15 min on ice. Cells were pelleted and resuspended in 1 ml HBSS + FBS (2%) and filtered twice through a 20 μm cell strainer (pluriSelect, KL-071912). Nuclei isolation was carried out as recommended by 10X Genomics by incubation in 100 µl of 0.1X lysis buffer (1 mM Tris-HCl pH7.4, 1 mM NaCl, 0.3 mM MgCl2, 0.1% BSA, 0.01% Tween-20, 0.01% NP40, 0.001% Digitonin (Invitrogen, BN2006), 0.1 mM DTT, 0.1 U/µl RNase inhibitor, in nuclease-free water) on ice for 5 min, followed by three washes and resuspension in 1 ml of chilled wash buffer (10 mM Tris-HCl pH7.4, 10 mM NaCl, 3 mM MgCl2, 1% BSA, 0.1% Tween-20, 1 mM DTT, 1 U/µl RNase inhibitor in nuclease-free water). Finally, nuclei were counted and resuspended in 1X nuclei buffer (provided in the 10x Genomics Single Cell Multiome ATAC Kit A; 1 mM DTT, 1 U/µl RNase inhibitor in nuclease free water) at a final concentration of approximately 2,000–3,000 nuclei/µl for sequencing on the 10X scMultiome platform.

### Single cell RNA-seq/ATAC-seq analysis

The scMultiome data analyzed in this paper was generated previously (GEO record number GSE223535; [17]) and the data analysis protocol is briefly summarized here. Fastq sequencing files from 10X Genomics multiomic single cell RNA-seq and ATAC-seq were processed through Cell Ranger ARC (v1.0.1) with a zebrafish GRCz11 library to obtain UMI gene expression counts and ATAC peak fragment counts. These were analyzed using the standard methods in the Signac (v1.6.0) package in R [19]. Gene expression was normalized with SCTransform and the dimensionality reduced with PCA. DNA accessibility was processed by performing latent semantic indexing. The Seurat weighted nearest neighbor method was used to compute a neighbor graph which was visualized with UMAP and clusters were annotated based on expression of marker genes. Per cell motif activities were scored with chromVar [20]. The Seurat Objects were integrated by finding the full intersecting ATAC peaks containing peaks in any of the three datasets and creating a new chromatin accessibility assay in each based on the full intersection peak file. The RNA-seq data was integrated with the Seurat V4 integration method and the new chromatin data integrated using Harmony followed by Seurat weighted nearest neighbor method to compute a UMAP and clusters. Dendrograms were calculated with the Seurat BuildClusterTree function. Volcano plots were generated with EnhancedVolcano v1.12.0 [21]. A biological replicate of HB13hpf multiomic 10X Genomics single cell library was generated and processed identically to the previous single cell libraries and the 2 replicates of HB13hpf were integrated and used to calculate GRNs using DIRECT-NET [22]. DIRECT-NET was written to analyze human single cell multiomic data for GRNs and relies on matching gene names in the data with JASPAR motif names. To utilize the DIRECT-NET package we first converted our scRNAseq gene names to their human orthologs using the human and zebrafish orthology file downloaded from https://zfin.org and modified the scripts slightly to accommodate the use of appropriate motif names. GRNs were visualized with Cytoscape [23] using the yfiles radial layout.

## Results

### Caudal hindbrain cells are molecularly identifiable at zebrafish segmentation stages

**Fig. 1:**
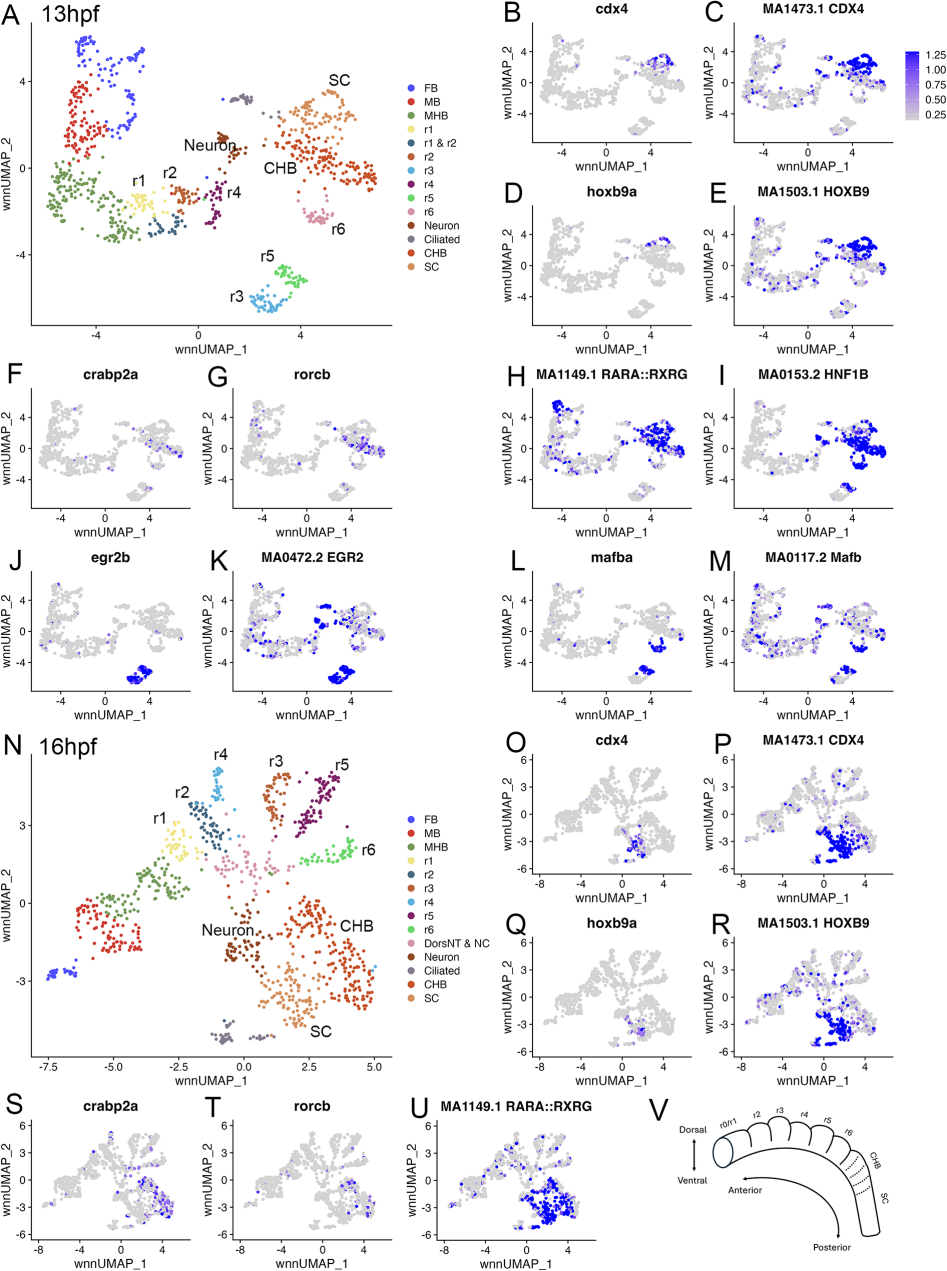
The caudal hindbrain is molecularly resolved at 13hpf and 16hpf in zebrafish. See also Additional file 1, Table S1. (**A**) UMAP of 13hpf neural clusters. (**B-M.**) Feature plots showing expressing of the indicated genes (**B**, **D**, **F**, **G**, **J**, **L**), or chromVar activity of the indicated TF motifs (**C**, **E**, **H**, **I**, **K**, **M**). (**N**) UMAP of 16hpf neural clusters. (**O-U.**) Feature plots showing expressing of the indicated genes (**O**, **Q**, **S**, **T**), or chromVar activity of the indicated TF motifs (**P**, **R**, **U**). (**V**) Schematic of zebrafish hindbrain with embryonic axes indicated. Dashed lines indicate boundaries delineating previously postulated pseudo-rhombomeres. UMAPs in A, N are based on 8.0 and 6.0 res clustering, respectively (chosen because this fully resolves r1-r6). In cases where multiple clusters were assigned the same identity, as was the case for FB, MB, MHB, CHB, and SC, they were combined into a single cluster labeled with that identity. Abbreviations used in all UMAPs: CHB - caudal hindbrain, HB - hindbrain, FB - forebrain, MB - midbrain, MHB - midbrain-hindbrain boundary, NT - neural tube, NC - neural crest, r - rhombomere, SC - spinal cord

We previously employed scMultiome analysis to simultaneously profile the transcriptional and chromatin states of individual neural progenitor cells from 10hpf (end of gastrulation; when neural marker genes are first expressed) to 16hpf (when the rhombomeres are becoming morphologically apparent) in zebrafish [17]. Our initial analysis focused on the region giving rise to hindbrain progenitors and we found that r1 through r6 are readily resolved at 13hpf and 16hpf [17]. Additionally, we detected two clusters that we provisionally identified as containing CHB and rostral SC cells, but that have not been examined in detail. In UMAPs containing the neural cluster subset from 13hpf and 16hpf zebrafish embryos, the provisional CHB and SC clusters are located adjacent to the r1-r6 clusters (Fig. 1A, N). By examining differentially expressed genes and differentially accessible transcription factor (TF) motifs (Additional file 1, Table S1), we find that one of these two clusters is characterized by expression of known SC markers, such as *cdx4* and *hoxb9a* [24–26] and shows a corresponding enrichment for accessible Hox and Cdx binding sites (Fig. 1B-E, O-R). We therefore conclude that this cluster corresponds to SC progenitors. The other cluster expresses *crabp2a* and *rorcb* (Fig. 1F, G, S, T) – two components of the RA-signaling pathway expressed in the caudal hindbrain at this stage [27]. This cluster is also distinct from the SC cluster in not expressing *cdx4* or *hoxb9* (Fig. 1B, D, O, Q) and from r5 and r6 in not expressing *egr2b* or *mafB* (restricted to r3/r5 and r5/r6, respectively; Fig. 1J, L). Accordingly, while this cluster shares available RAR: RXR and Hnf1b motifs with the SC cluster (Fig. 1H, I, U), it displays a unique combination of accessible TF binding sites, such that it largely lacks accessible binding sites for Egr2 and MafB (Fig. 1K, M), as well as for Cdx4 and Hoxb9 (Fig. 1C, E, P, R). We conclude that this cluster corresponds to the CHB and that the CHB is molecularly distinct from r6 and the SC at both 13hpf and 16hpf (Fig. 1V).

### The embryonic caudal hindbrain contains cells of a single anteroposterior molecular identity

**Fig. 2:**
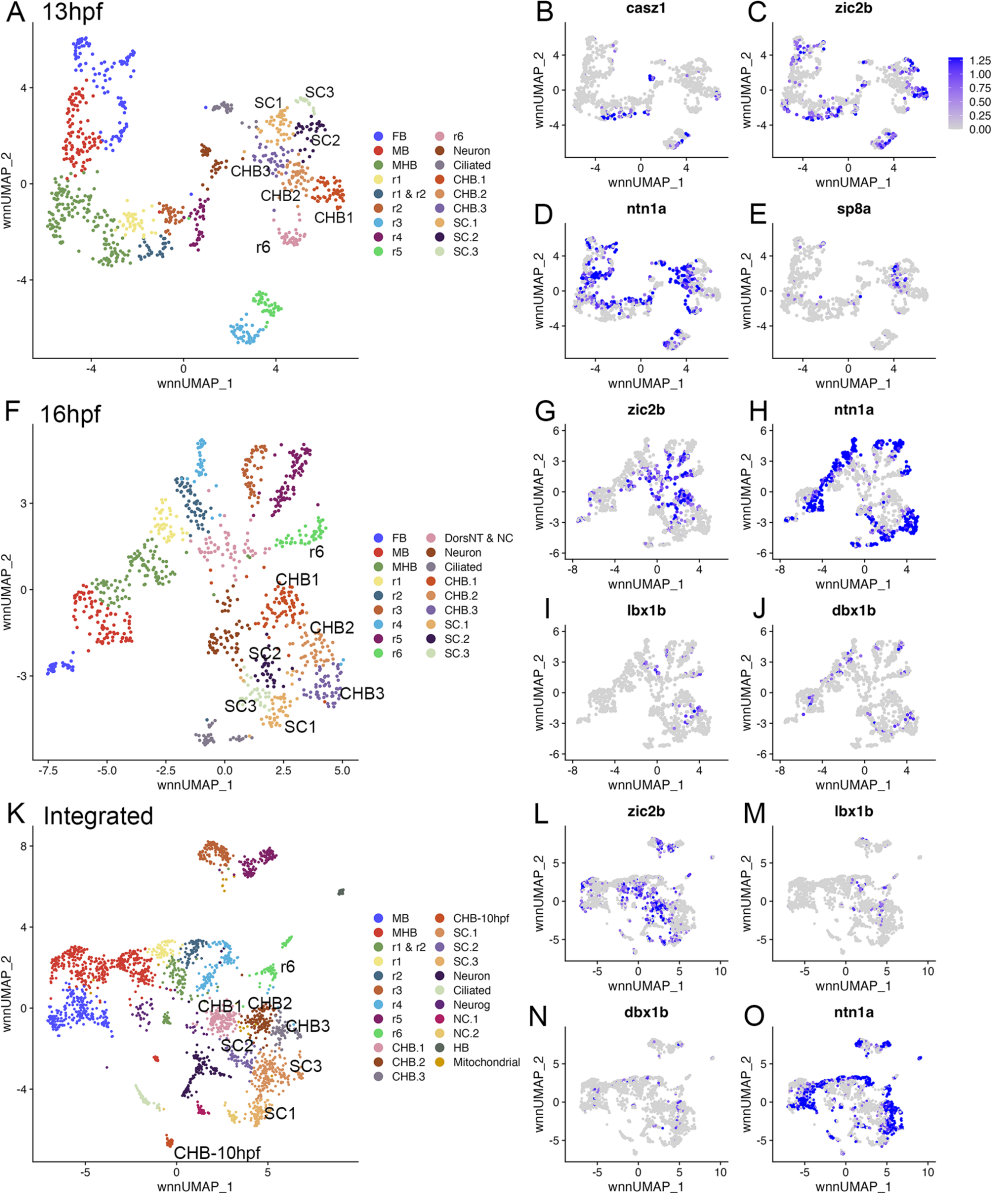
The caudal hindbrain can be subdivided along its dorsoventral axis. See also Additional file 2, Table S2. (**A**) UMAP of 13hpf neural clusters. (**B-E**) Feature plots showing expressing of the indicated genes. (**F**) UMAP of 16hpf neural clusters. (**G-J**) Feature plots showing expressing of the indicated genes. (**K**) UMAP of integrated 10hpf, 13hpf and 16hpf neural clusters. (**L-O**) Feature plots showing expressing of the indicated genes. UMAPs in A, F, K are based on 8.0, 6.0 and 5.0 res clustering, respectively (chosen because this fully resolves r1-r6). In cases where multiple clusters were assigned the same identity, they were combined into a single cluster with that identity – except for CHB and SC clusters that were left separate and labeled consecutively. See legend to Fig. 1 for abbreviations

To assess whether the CHB contains one or more rhombomere-like progenitor populations along its AP axis, we examined the CHB cluster further. We find that increasing the resolution of the 13hpf UMAP reveals three sub clusters for the CHB (Fig. 2A). To determine if these subclusters correspond to distinct rhombomere-like structures, we identified genes enriched in each subcluster relative to all other clusters in the UMAP (Additional file 2, Table S2). We find that clusters CHB.1 and CHB.3 are enriched for dorsal and ventral genes, respectively, such that cells in cluster CHB.1 express *casz1* and *zic* family TFs (Fig. 2B, C), whereas CHB.3 cells express *ntn1a* and *sp8a* (Fig. 2D, E). We observe the same pattern in the 16hpf UMAP, where increasing the resolution also identifies three CHB clusters (Fig. 2F; Additional file 2, Table S2) with CHB.1 expressing dorsal (*zic2b*; Fig. 2G) and CHB.3 expressing ventral (*ntn1a*; Fig. 2H) genes. We additionally find that the CHB.2 cluster expresses *lbx1b* and *dbx1b*, two markers of neural progenitors arising at medial positions of the neural tube [28–30], at 16hpf (Fig. 2I, J). To confirm the identities of these CHB clusters, we integrated data across the 10hpf, 13hpf and 16hpf timepoints, projected it as an integrated UMAP (Fig. 2K; Additional file 2, Table S2) and examined gene expression. The integrated UMAP again shows three CHB subclusters (with a fourth CHB-like cluster containing only 10hpf cells that appear too immature to be computationally assigned to one of the more mature CHB clusters). We again find that *lbx1b* and *dbx1b* expressing cells are present in the CHB.2 cluster located between *zic2b* (CHB.1) and *ntn1a* (CHB.3) expressing cells (Fig. 2L-O) and note that the SC similarly resolves into three clusters. These analyses identified three CHB clusters corresponding to progenitors positioned along the dorsoventral axis of the neural tube but did not detect progenitor clusters with distinct AP molecular identities within the CHB.

**Fig. 3:**
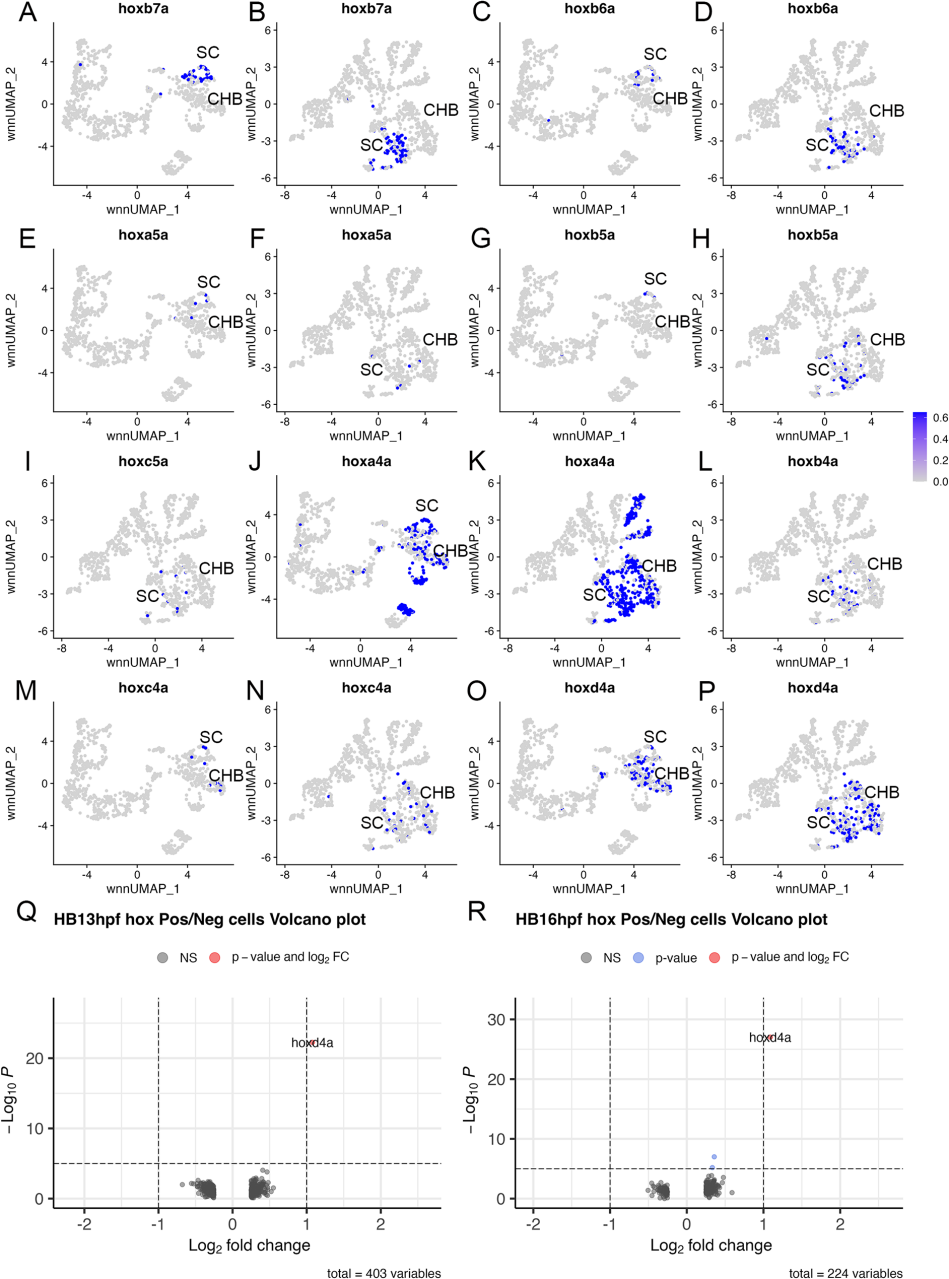
The caudal hindbrain does not contain pseudo-rhombomeres. See also Additional file 3, Table S3. (**A-P**) Feature plots showing expressing of the indicated *hox* genes at 13hpf (**A**, **C**, **E**, **G**, **J**, **M**, **O**) or 16hpf (**B**, **D**, **F**, **H**, **I**, **K**, **L**, **N**, **P**). **Q, R.** Volcano plots showing genes differentially expressed between PG4 *hox-*positive and PG4 *hox-*negative cells at 13hpf (**Q**) or 16hpf (**R**). A positive log2FC indicates higher expression in PG4 positive cells. See legend to Fig. 1 for abbreviations

As an alternative approach to determine if rhombomere-like structures are present along the AP axis in the early CHB, we assessed the expression of genes previously reported to delineate such domains – particularly *hox* genes. Analyses in chick and mouse report that *hox* genes have distinct expression boundaries within the CHB [9, 10] and these findings have been used to postulate the presence of pseudo-rhombomeres (PRs) in the CHB. While *hox* genes are expressed within the CHB in zebrafish [7, 25, 31], we find that several *hox* genes postulated to demarcate PRs in the CHB are expressed exclusively in the spinal cord at both 13hpf and 16hpf. Accordingly, *hoxb7* is reported to define the boundary between PR10 and PR11, while *hoxa6* and *hoxb6* are proposed to delineate PR9 from PR10 [10], but we find expression of zebrafish PG6 and PG7 *hox* genes to be within the SC at both 13hpf and 16hpf (Fig. 3A-D). Similarly, *hoxa5*, *hoxb5* and *hoxc5* are proposed to delineate PR8 from PR9 [10], but we find that zebrafish PG5 *hox* genes are expressed at low levels at 13hpf and 16hpf and their expression is not restricted to the CHB (Fig. 3E-I). These data demonstrate that PG5-7 *hox* genes are not expressed in distinct CHB clusters and are not compatible with the presence of PRs at 13hpf or 16hpf in the zebrafish CHB. PG4 *hox* genes – that are reported to delineate PR7 from PR8 [10] – display varied expression within the zebrafish CHB. While *hoxa4a* has its anterior border at the r4/r5 boundary (Fig. 3J, K; [31]), *hoxb4a*, *hoxc4a* and *hoxd4a* are expressed in subsets of CHB cells to various degrees (Fig. 3L-P), but these cells do not form clear clusters. To determine if CHB cells with divergent PG4 *hox* expression profiles possess additional molecular features that might distinguish them as PRs, we computationally selected all CHB cells that express *hoxb4a, hoxc4a* or *hoxd4a*. We then compared the gene expression profile of these CHB cells to those that do not express *hoxb4a, hoxc4a* or *hoxd4a* and generated volcano plots (Fig. 3Q, R; Additional file 3, Table S3). This analysis did not identify any other genes that are significantly restricted to PG4 *hox* expressing cells. Based on these analyses, we do not detect a molecular subdivision of the zebrafish CHB into multiple rhombomere-like structures at 13hpf or 16hpf – embryonic stages when r1-r6 are already well established – but we cannot exclude the possibility that such structures form in other species or at later stages of neural development.

### The embryonic caudal hindbrain shares gene expression with both r6 and spinal cord, but is defined by a unique gene regulatory network

**Fig. 4:**
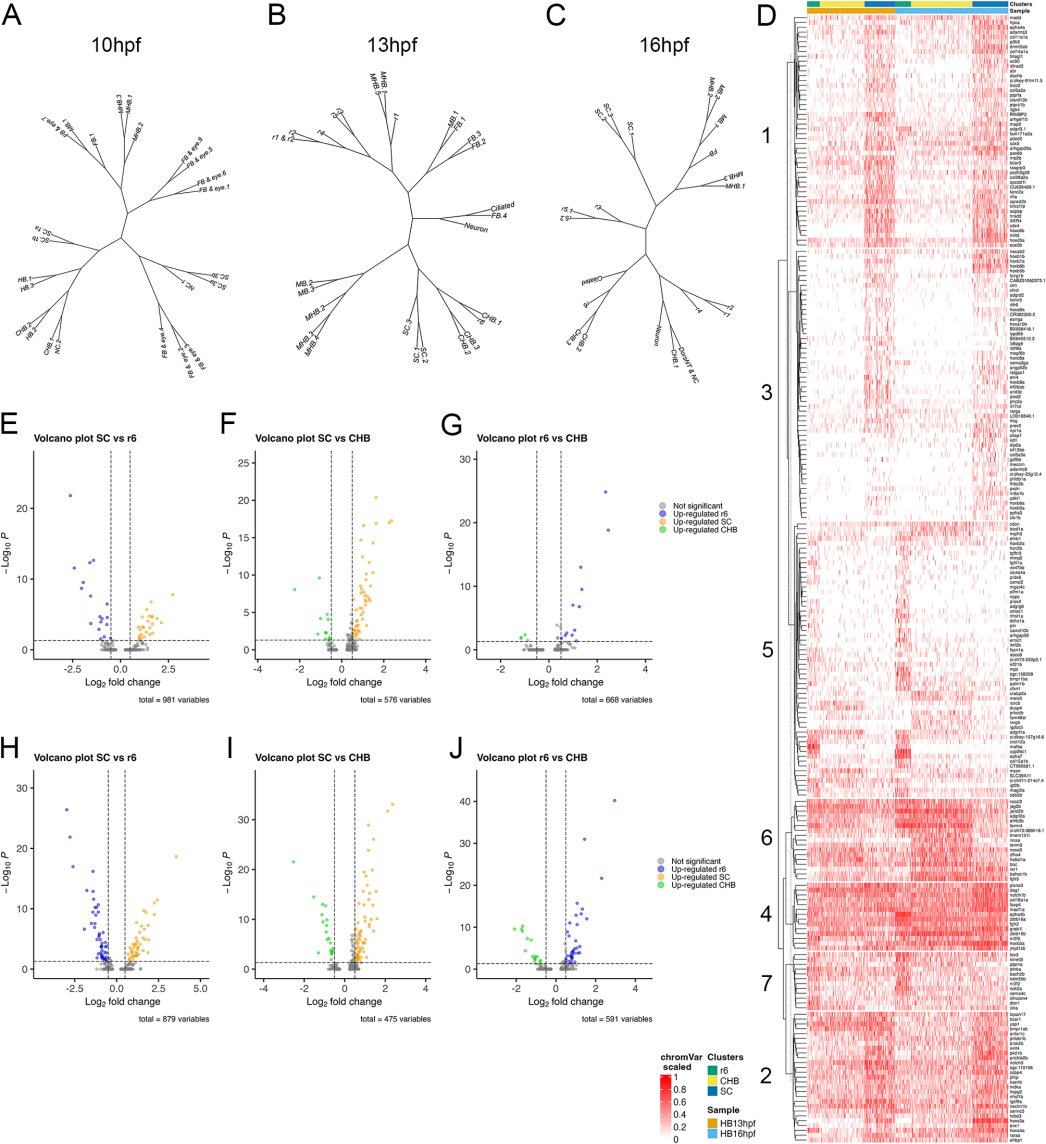
The caudal hindbrain shares gene expression with rhombomere 6 and spinal cord. See also Additional file 6, Table S5 and Additional file 5, Figure S1. **A-C.** Dendrograms showing relationship between neural clusters at 10hpf (**A**), 13hpf (**B**) and 16hpf (**C**). The clusters used for the dendrograms are based on res 8.0 (10hpf), res 8.0 (13hpf), and res 6.0 (16hpf) – chosen because this fully resolves r1-r6 – with all resulting clusters left separate. (**D**) Heatmap displaying genes differentially expressed among r6, CHB and SC clustered using Ward D2. (**E-J**) Volcano plots showing genes differentially expressed in the indicated pairwise comparison among r6, CHB and SC at 13hpf (E-G) or 16hpf (H-J). The cluster definitions for the heatmap and the volcano plots are the same as in Fig. 1A, N. See legend to Fig. 1 for abbreviations

The anatomical location of the CHB between r6 and the SC raises the question how closely related the CHB is to these adjacent neural structures. We find that CHB cells cluster near SC cells in the 13hpf and 16hpf UMAPs (Fig. 1A, N), but proximity in the UMAP is not a reliable indicator of the relationship between two cell populations. We therefore used the full gene expression profiles for the r6, CHB and SC clusters, and projected the data as dendrograms (Fig. 4A-C). This analysis shows the CHB clusters sharing branches more closely with r6 than SC both at 13hpf and 16hpf. To further explore this finding, we generated heatmaps of genes differentially expressed in each cluster relative to the other two clusters for r6, CHB and SC at 13hpf and 16hpf (Additional file 4, Table S4; Additional file 5, Figure S1A-C). In this manner, we identified numerous genes differentially expressed in r6 and the SC, and we note an apparent trend towards more differentially expressed genes being present at 16hpf relative to 13hpf (Additional file 5, Figure S1A-C). In contrast, there are relatively few genes differentially expressed in the CHB cluster and many of these are also expressed at comparatively high levels in r6 – particularly at 13hpf (Additional file 5, Figure S1B). We next combined the differentially expressed genes from each cluster at 13hpf and 16hpf, projected their expression in a single heatmap, and used Ward D2 clustering to group them based on similarities in expression patterns (Fig. 4D). This analysis identified groups of genes with enriched expression in the SC (Groups 1 and 3 strongly enriched; Group 2 weakly enriched) or r6 (Group 5 strongly enriched; Group 7 weakly enriched). In contrast, only a single group of genes (Group 6) shows enriched expression in the CHB and most of these genes are also expressed in r6 or the SC – again with a trend towards greater similarity between the CHB and r6 at 13hpf. Accordingly, pairwise comparisons of gene expression between clusters revealed fewer genes enriched in the CHB relative to r6 than relative to the SC at 13hpf (Additional file 6, Table S5; Fig. 4E-G), with the number of CHB-enriched genes increasing by 16hpf (Fig. 4H-J). This analysis demonstrates that, while r6 and the SC are characterized by unique gene expression profiles, the gene expression profile of the CHB overlaps extensively with r6 and, to lesser extent, with the SC.

**Fig. 5:**
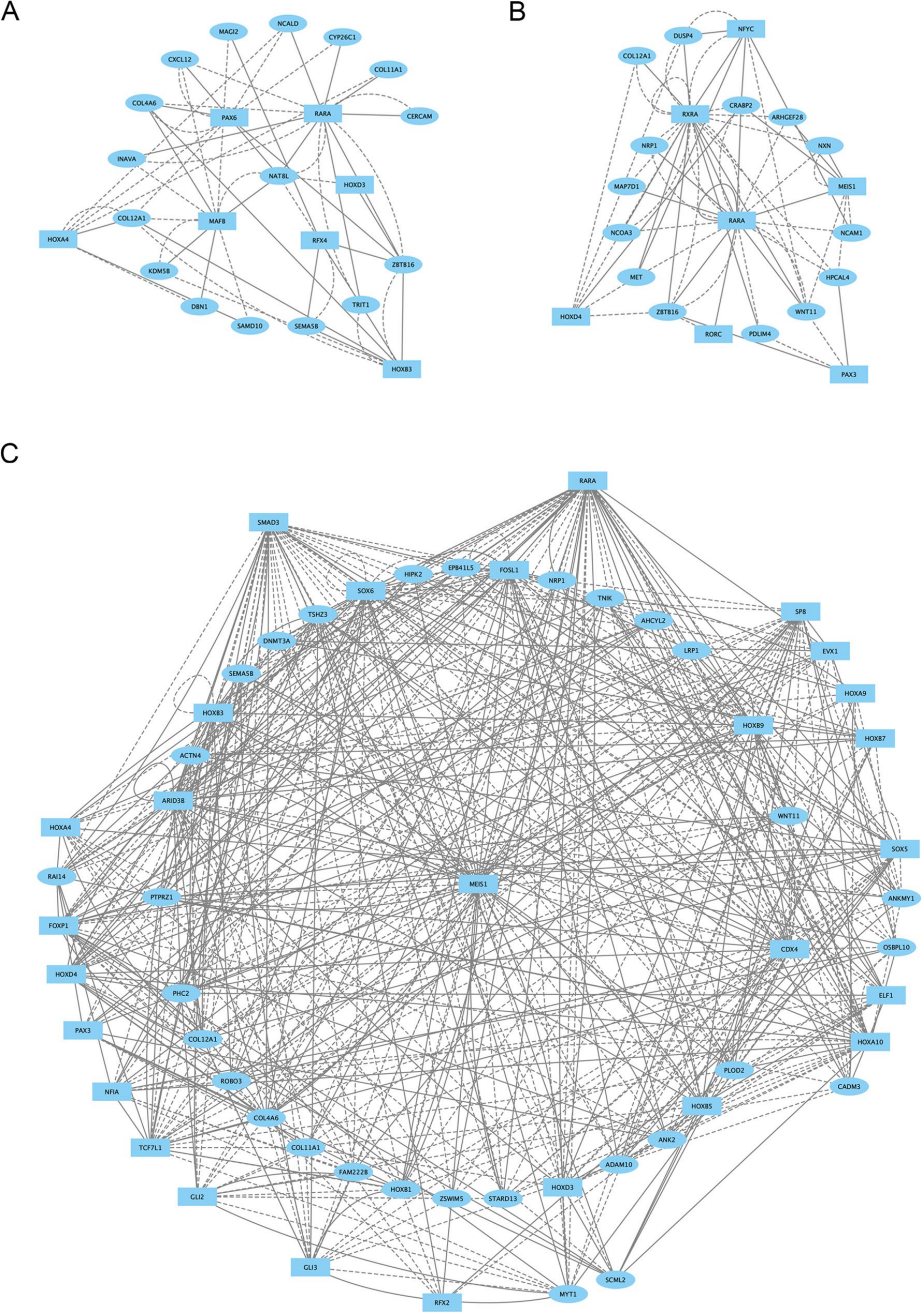
Caudal hindbrain cells display a distinct gene regulatory network at 13hpf. (**A-C.**) Direct-Net derived gene regulatory networks for r6 (**A**), CHB (**B**) and SC (**C**) at 13hpf. TFs (squares) and non-TFs (ovals) are linked by the presence of accessible TF motifs within 500 bp (solid lines) or 250 kb (dashed lines) of a gene’s transcription start site. Due to the large network produced for SC, only nodes with more than ten connections are shown (the full SC network is shown in Additional file 7, Figure S2B)

To compare the genetic programs underlying the differential gene expression in each cell population, we used the differentially expressed genes and differentially accessible chromatin regions for each cluster relative to all other neural clusters at 13hpf (Additional file 1, Table S1) and employed the DirectNet tool [22] to generate GRNs for each cluster (Fig. 5A-C; Additional file 7, Figure S2B). We find that these GRNs include TFs previously reported in each population, such that the *mafb* and *cdx4* TFs occupy central positions in the r6 and SC GRNs, respectively (as reported previously; [26, 32, 33]). We also note that the GRNs share components – e.g., PG4 *hox* genes are present in all three GRNs, in accordance with their reported expression patterns [31] – indicating that these GRNs accurately capture bona fide regulatory interactions. To determine how the GRNs compare between the clusters, we compared the components of each GRN in a Venn diagram (Additional file 7, Figure S2A) and identified genes that are shared or distinct between them. Although the SC GRN is substantially more complex than the r6 or CHB GRNs, we find that each cluster is defined by a GRN that is largely unique to that cluster. Indeed, although the CHB shares extensive gene expression with both r6 and the SC, it is nevertheless characterized by a unique GRN, demonstrating that r6, CHB and SC represent molecularly distinct cell populations with unique gene regulatory networks and gene expression profiles.

### Late gastrula stage caudal hindbrain progenitors are more closely related to r6 cells

**Fig. 6:**
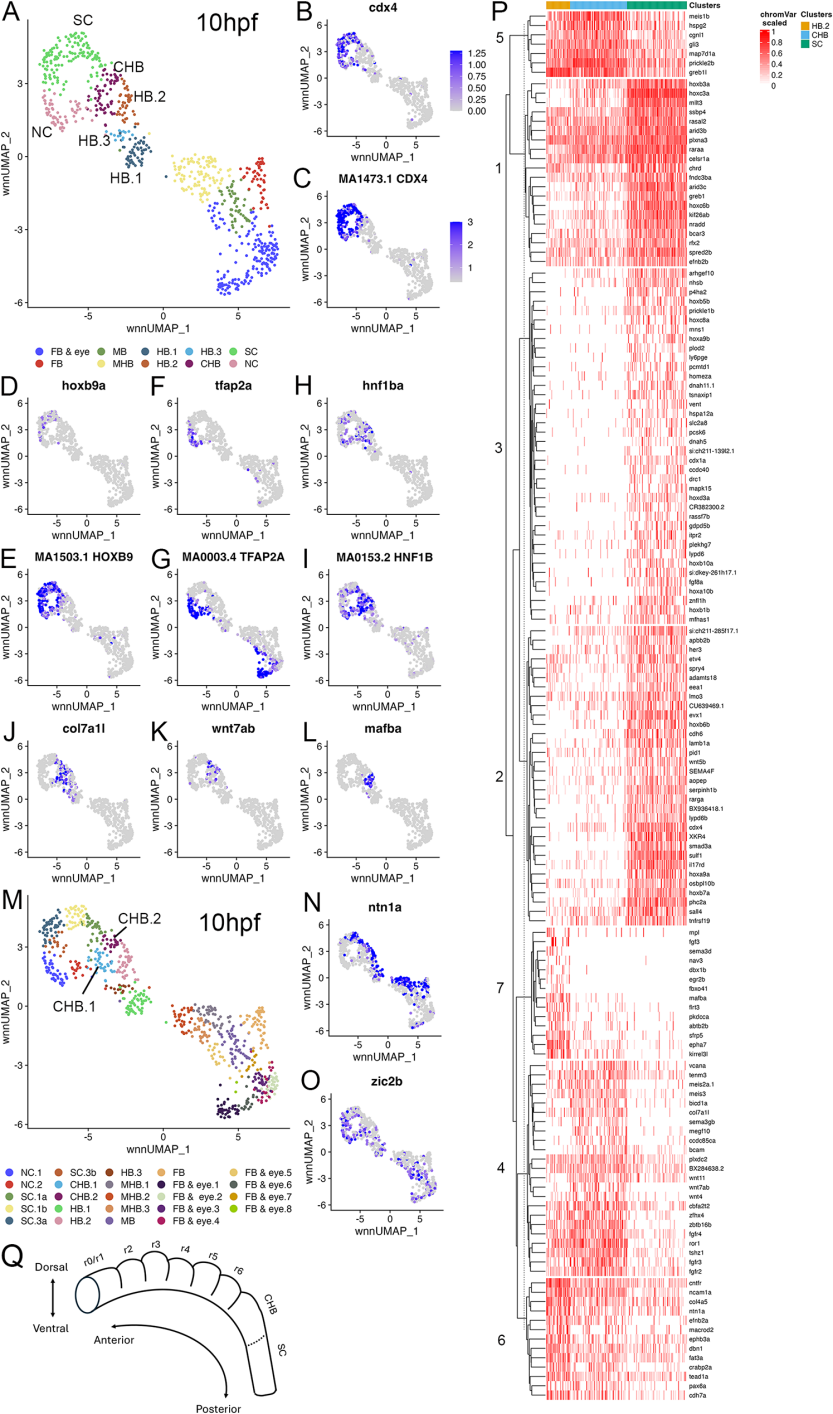
Late gastrula stage caudal hindbrain progenitors are closely related to r6 cells. See also Additional file 8, Table S6 and Additional file 10, Figure S3. (**A**) UMAP of 10hpf neural clusters. (**B-L**) Feature plots showing expressing of the indicated genes (**B**, **D**, **F**, **H**, **J**, **K**, **L**), or chromVar activity of the indicated TF motifs (**C**, **E**, **G**, **I**). (**M**) UMAP of 10hpf neural clusters. (**N, O**) Feature plots showing expressing of the indicated gene. (**P**) Heatmap displaying genes differentially expressed among HB.2, CHB and SC clustered using Ward D2. (**Q**) Schematic of zebrafish hindbrain based on scMultiome analysis. Dashed line indicates boundary between CHB and SC. UMAPs in A, M are based on 8.0 res clustering (chosen because this resolves HB.1-HB.3). In A, multiple clusters assigned the same identity were combined into a single cluster labeled with that identity, except for the HB clusters. In M, all clusters were left separate and labeled consecutively. See legend to Fig. 1 for abbreviations

Our finding that the CHB shares more gene expression with r6 than SC at 13hpf and 16hpf raises the question if this is the case also earlier in embryogenesis. To address this possibility, we examined scMultiome data from embryos at the end of gastrulation (10hpf). As we reported previously [17], individual rhombomeres have not yet formed at this stage. Instead, rhombomere progenitors are found in three clusters (HB.1-HB.3) and we observe three UMAP clusters adjacent to HB.1-HB.3 (Additional file 8, Table S6; Fig. 6A). One cluster expresses *cdx4* and *hoxb9a* and is enriched for the corresponding TF binding motifs (Fig. 6B-E), identifying this cluster as containing SC progenitors. A second cluster is enriched for *tfap2a* expression and the Tfap2a binding motif, while also expressing *cdx4* (Fig. 6F, G). Since *tfap2a* encodes a TF involved in neural crest (NC) formation [34, 35], we conclude that this cluster corresponds to early NC progenitors. The third cluster shares enrichment of *hnf1ba* expression with adjacent clusters (Fig. 6H, I), but lacks expression of SC (*cdx4* and *hoxb9a*) and NC (*tfap2a*) markers (Fig. 6B-E) and is unique in its expression of *col7a1l* and *wnt7ab* (Fig. 6J, K). At higher resolution (Additional file 8, Table S6; Fig. 6M), this cluster becomes further resolved into ventral and dorsal subclusters as indicated by expression of *ntn1a* and *zic2b*, respectively (Fig. 6N, O). We conclude that this third cluster corresponds to late gastrula CHB progenitors.

We note that the late gastrula CHB cluster is located immediately adjacent to the HB.2 cluster (that contains r5 and r6 progenitors; [17]) but differs from it by lacking expression of the *mafba* r5/r6 marker (Fig. 6L). Accordingly, we also find that the CHB clusters are located on a branch together with the HB.2 cluster at this stage (Fig. 4A). To better understand the relationship between HB.2, CHB and SC at 10hpf, we next identified genes differentially expressed in each cluster relative to the other two and projected their expression in heatmaps (Additional file 9, Table S7; Additional file 10, Supplemental figure S3A-C). We find that HB.2 and SC possess distinct gene expression profiles, but the CHB shares extensive expression with the HB.2 cluster. When we combine the differentially expressed genes from each cluster, project their expression in a single heatmap, and use Ward D2 clustering to group them based on similarities in expression patterns (Fig. 6P), we again find that HB.2 (Groups 6 and 7) and SC (Groups 1–3) uniquely express several genes, while the CHB (Groups 4 and 5) shares extensive gene expression with HB.2. Indeed, pairwise comparisons of gene expression between clusters revealed that only a few genes are enriched in the CHB relative to HB.2 at 10hpf, whereas there are numerous genes differentially expressed between CHB and SC at this stage (Additional file 11, Table S8; Additional file 10, Supplemental figure S3D-F). Together with the data in Fig. 4, this analysis demonstrates that CHB progenitors are highly related to HB.2 and r6 progenitors at early stages of embryogenesis and that these populations become more distinct from each other as development progresses.

## Discussion

The neuromere model postulates that the developing CNS is transiently divided into transverse compartments that serve to position neural progenitors along the embryonic AP axis [3, 36]. Such neuromeres are particularly evident in the embryonic hindbrain, which is clearly demarcated at its rostral end by a morphological constriction at the midbrain-hindbrain boundary. The embryonic hindbrain is divided into six anterior rhombomeres (r1-r6) separated by morphological boundaries that can be transiently observed visually. The region of the hindbrain caudal to r6 is less well defined and, in contrast to the midbrain-hindbrain boundary, there is no morphologically apparent hindbrain-spinal cord boundary. There have been prior suggestions that the region caudal to r6 contains additional rhombomeres, but there is disagreement as to their existence and the potential number of such caudal rhombomeres. For instance, diagrams based on visual observations in mouse and chick often include one additional rhombomere boundary caudal to r6 that would delineate r7 from r8, with r8 then abutting the spinal cord [5, 7]. More recently, additional rhombomere-like structures have been proposed in the murine caudal hindbrain based on fate mapping experiments – where transplants derived from specific anteroposterior levels were found to give rise to motor and sensory nuclei corresponding to their anteroposterior origin [11] – bringing the potential total number of rhombomeres to eleven. Notably, these caudal rhombomeres do not seem to be demarcated by boundaries analogous to those defining r1-r6, and the caudal rhombomeres are therefore referred to as crypto- or pseudo-rhombomeres [9–11]. Similarly, the zebrafish hindbrain contains six well-defined anterior rhombomeres, but investigators disagree on the presence of more caudal rhombomeres with some identifying one (corresponding to r7; [37]), while others propose as many as three (r7-r9; [38]).

Rhombomeres 1 to 6 are molecularly defined by numerous differentially expressed genes, as well as by distinct chromatin accessibility profiles, that establish unique GRNs for each rhombomere [7, 17]. Members of the *hox* family of transcriptional regulators are particularly important in this regard, as their expression patterns coincide with specific rhombomeres, and disruptions of *hox* gene function lead to defects in rhombomere formation (reviewed in [7]). Analogous to the situation in r1-r6, analyses of *hox* gene expression in the CHB of mouse and chick embryos have been used to postulate the existence of pseudo-rhombomeres, but it is not clear if pseudo-rhombomeres can be defined by additional molecular features. Importantly, while the pseudo-rhombomeres lack morphologically distinct boundaries, they would nevertheless be expected to be molecularly distinct from each other – since each pseudo-rhombomere is predicted to give rise to specific motor and sensory nuclei of the mature brainstem [11]. Specifically, it would be expected that each pseudo-rhombomere is defined by a unique genetic program to support the formation of such neural derivatives and this should manifest itself in a unique GRN for each pseudo-rhombomere, as is seen for r1-r6 [17]. However, a lack of molecular data for the developing caudal hindbrain has made it difficult to address this comprehensively. We recently overcame this problem using scMultiome analysis, which allowed us to resolve and generate putative GRNs for r1-r6 [17]. Our current analysis identified a population of cells that corresponds to the caudal hindbrain. Increasing the resolution of our analysis identified dorsal, medial, and ventral progenitor populations within this caudal hindbrain population, but did not detect molecularly distinct subpopulations that might correspond to pseudo-rhombomeres. We also note that r1-r6 can be resolved by our analysis both at 13hpf (early somitogenesis) and 16hpf (mid-somitogenesis; when r1-r6 are visually apparent), but the CHB population was not resolved into pseudo-rhombomeres in UMAPs at either stage. While it could be argued that our analysis is incapable of resolving populations that lack a morphologically apparent border, we note that the caudal hindbrain is clearly distinct from the spinal cord in our UMAPs, dendrograms, and GRNs, demonstrating that our analyses can distinguish progenitor populations not separated by visual borders. While it remains possible that pseudo-rhombomeres form later than more rostral rhombomeres, neuronal differentiation is initiated by 16hpf, and – since the rhombomeres are thought to direct the formation of neural structures – it would have been expected for pseudo-rhombomeres to form prior to the onset of neuronal differentiation, as is the case for r1-r6.

The CHB is also unique in that it has a distinct border with r6, but no clear border with the spinal cord. In addition, this region of the CNS represents the transition between neural structures derived from the neural plate (forebrain, midbrain, hindbrain and rostral spinal cord) and those derived from neuromesodermal progenitors (NMPs; [39–41]), such as the caudal spinal cord. It is therefore unclear if the CHB is more closely related to r6 or the spinal cord, and if it shares a closer embryonic origin with either structure. In the absence of more extensive gene expression data, it has been difficult to directly address whether the CHB gene expression program is more closely related to r6 or to the SC. Using the comprehensive gene expression profiles derived from our scMultiome analyses, we were able to carry out specific comparisons between these structures at multiple stages. We find that r6 and the SC are clearly distinct at all stages analyzed. In contrast, while the CHB is clearly distinct from the SC, it shares considerable gene expression overlap with r6. This overlap was more pronounced at earlier stages of development, such that the r6 and CHB similarity decreased from 10hpf to 16hpf.

## Conclusions

Our molecular data indicate that the zebrafish CHB represents a single neuromere compartment at developmental stages when the anterior rhombomeres 1–6 have already formed (Fig. 6Q). While it remains possible that pseudo-rhombomeres form in the CHB at later stages, the fact that neural differentiation is already underway at these timepoints argues against this possibility. We also demonstrate that the CHB transcriptional profile is more closely related to r6 than to the spinal cord at the earliest stages analyzed, indicating that r6 and the CHB may share an embryonic origin.

### Electronic supplementary material

Below is the link to the electronic supplementary material.

Additional File 1: Table S1

Additional File 2: Table S2

Additional File 3: Table S3

Additional File 4: Table S4

Additional File 5: Figure S1

Additional File 6: Table S5

Additional File 7: Figure S2

Additional File 8: Table S6

Additional File 9: Table S7

Additional File 10: Figure S3

Additional File 11: Table S8

Additional File 12. Supplemental legends

## Data Availability

The datasets generated and/or analyzed in the current study are available in the accompanying Additional Files (Tables S1-S8) and in the GEO repository under record number GSE223535. The code used to generate the data for each figure is available at https://github.com/rebeccaorourke-cu/Sagerstrom_CHB_SC_r6.

## Abbreviations

CHB: Caudal hindbrain
HB: Hindbrain
FB: Forebrain
MB: Midbrain
MHB: Midbrain-hindbrain boundary
NT: Neural tube
NC: Neural crest
r: Rhombomere
SC: spinal cord
