# Correction: scMultiome analysis identifies a single caudal hindbrain compartment in the developing zebrafish nervous system

**DOI:** 10.1186/s13064-024-00192-4

**Published:** 2024-08-03

**Authors:** Jessica Warns, Yong-II Kim, Rebecca O’Rourke, Charles G. Sagerström

**Affiliations:** 1 Section of Developmental Biology, Department of Pediatrics, University of Colorado Medical School, 12801 E. 17th Avenue, Aurora, CO 80045 USA; 2 Department of Science and Math, Northern State University, 1200 S. Jay St, Aberdeen, SD 57401 USA

**Correction: Neural Dev 19, 12 (2024)**

10.1186/s13064-024-00189-z

Following publication of the original article [1], the author reported errors in the additional file:

1. In the supplementary information section, the caption ‘Supplementary Material’ should be change to ‘Additional file’.

2. The additional files are posted in the incorrect order. See below table for the correct order and additional file captions

**Table:** 

Incorrect captions	Correct captions
Supplementary Material 1	Additional File 1: Table S1
Supplementary Material 2	Additional File 2: Table S2
Supplementary Material 3	Additional File 3: Table S3
Supplementary Material 4	Additional File 4: Table S4
Supplementary Material 5	Additional File 5: Figure S1
Supplementary Material 6	Additional File 6: Table S5
Supplementary Material 7	Additional File 7: Figure S2
Supplementary Material 8	Additional File 8: Table S6
Supplementary Material 9	Additional File 9: Table S7
Supplementary Material 10	Additional File 10: Figure S3
Supplementary Material 11	Additional File 11: Table S8

3. The legend/description of the additional files were missing. The missing additional file legends/descriptions were included as Additional File 12. Supplemental legends in this correction article.

The original article [1] has been corrected.

### Supplementary Information

Additional file 1: Table S1

Additional file 2: Table S2

Additional file 3: Table S3

Additional file 4: Table S4

Additional file 5: Figure S1

Additional file 6: Table S5

Additional file 7: Figure S2

Additional file 8: Table S6

Additional file 9: Table S7

Additional file 10: Figure S3

Additional file 11: Table S8

Additional file 12: Supplemental legends
