# Supplementary figures and images for: scMultiome analysis identifies a single caudal hindbrain compartment in the developing zebrafish nervous system
Source: Neural Dev. 2024 Jul 5;19:12. doi: 10.1186/s13064-024-00189-z (PMC11225431; doi:10.1186/s13064-024-00189-z)

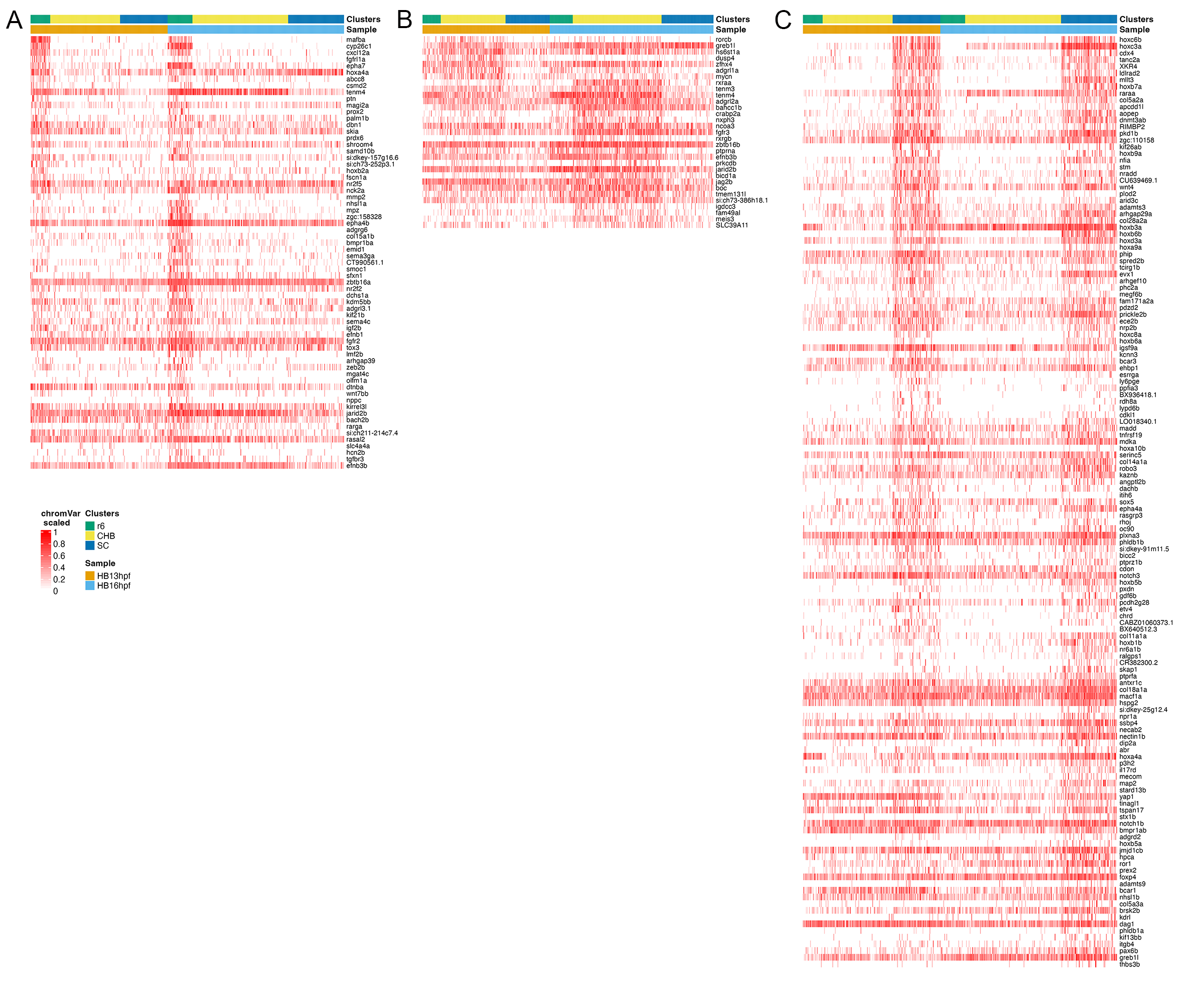

Supplement: Supplementary file 5 — Additional File 5: Figure S1 [file 13064_2024_189_MOESM5_ESM.tif]

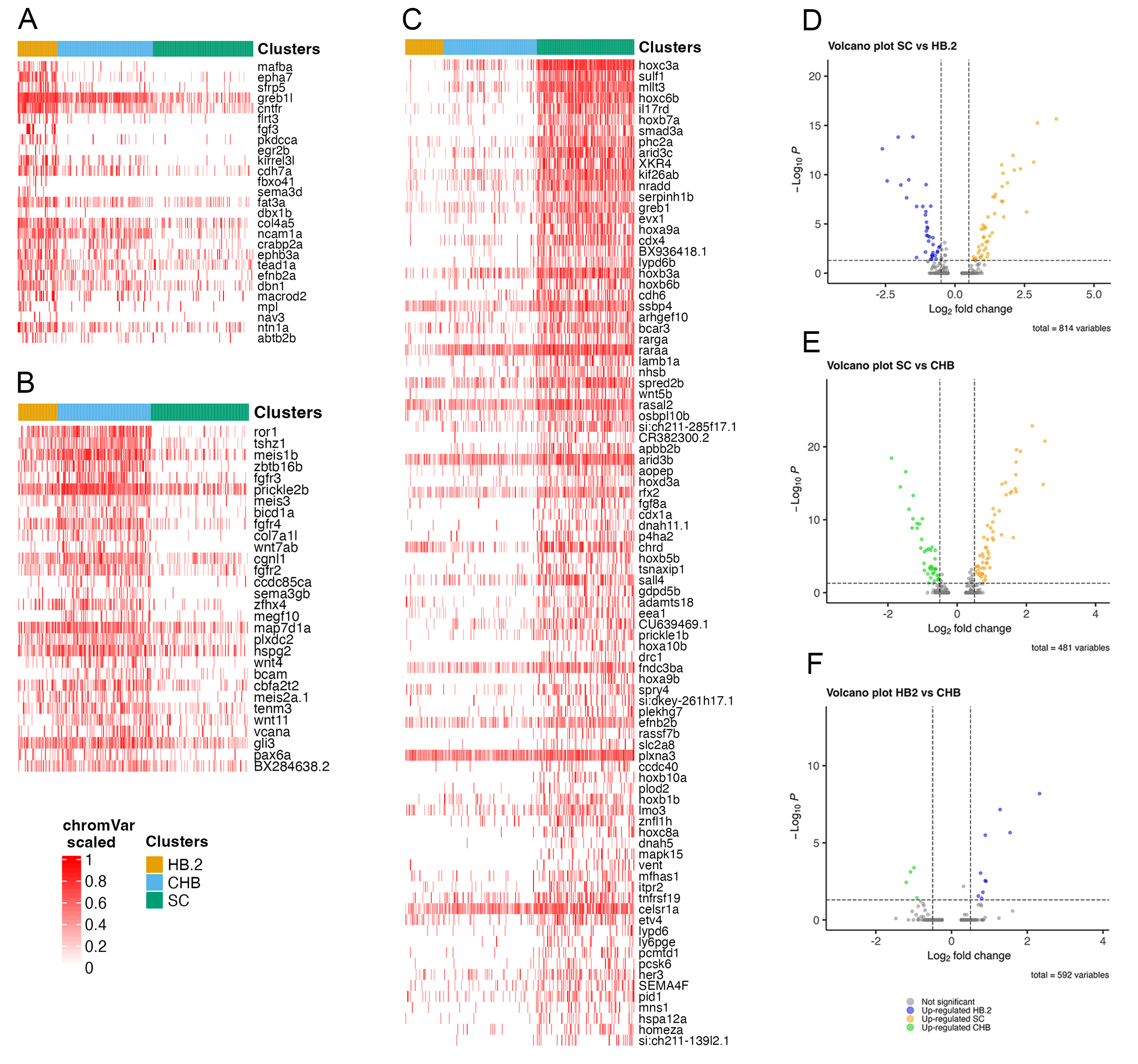

Supplement: Supplementary file 10 — Additional File 10: Figure S3 [file 13064_2024_189_MOESM10_ESM.tif]
