# Supplementary material for: scMultiome analysis identifies a single caudal hindbrain compartment in the developing zebrafish nervous system
Source: Neural Dev. 2024 Jul 5;19:12. doi: 10.1186/s13064-024-00189-z (PMC11225431; doi:10.1186/s13064-024-00189-z)
Supplement: Supplementary file 12 — Additional File 12. Supplemental legends [file 13064_2024_189_MOESM12_ESM.docx]

**Additional files**

Additional file 1, Table S1.

Table S1 is associated with Figure 1 and lists all genes and TF motifs differentially enriched in each cluster relative to all clusters present in that analysis.

Additional file 2, Table S2.

Table S2 is associated with Figure 2 and lists all genes differentially expressed in each cluster relative to all clusters present in that analysis.

Additional file 3, Table S3.

Table S3 is associated with Figure 3Q, R and lists all genes differentially expressed in PG4 *hox*-expressing versus PG4-non-*hox* expressing CHB cells.

Additional file 4, Table S4.

Table S4 is associated with Figure S1 and lists all genes differentially expressed in one cluster relative to the other two among r6, CHB and SC.

Additional file 5, Figure S1. The caudal hindbrain shares gene expression with rhombomere 6 and spinal cord. **A-C.** Heatmaps displaying genes enriched in r6 (A), CHB (B) or SC (C) relative to the other two clusters at the indicated stage. The cluster definitions used for the heatmaps are the same as in Figure 1A, N. See legend to figure 1 for abbreviations.

Additional file 6, Table S5.

Table S5 is associated with Figure 4E-J and lists all genes differentially expressed between two clusters (among r6, CHB and SC).

Additional file 7, Figure S2. Caudal hindbrain cells display a distinct gene regulatory network at 13hpf. **A.** Venn diagram indicating components that are shared, or distinct, between the gene regulatory networks for r6, CHB and SC. **B.** Direct-Net derived gene regulatory network for SC at 13hpf with all connections shown. TFs (squares) and non-TFs (ovals) are linked by the presence of accessible TF motifs within 500bp (solid lines) or 250kb (dashed lines) of a gene’s transcription start site. See legend to figure 1 for abbreviations.

Additional file 8, Table S6.

Table S6 is associated with Figure 6A, M and lists all genes and TF motifs differentially enriched in each cluster relative to all clusters present in that analysis.

Additional file 9, Table S7.

Table S7 is associated with Figure S3A-C and lists all genes differentially expressed in one cluster relative to the other two (among HB.2, CHB and SC).

Additional file 10, Figure S3. Late gastrula stage caudal hindbrain progenitors are more closely related to r6 cells. **A-C.** Heatmaps displaying genes enriched in HB.2 (A), CHB (B) or SC (C) relative to the other two clusters at 10hpf. D-F. Volcano plots showing genes differentially expressed in the indicated pairwise comparison among HB.2, CHB and SC at 10hpf. The cluster definitions for the heatmaps and volcano plots are the same as in Figure 6A. See legend to figure 1 for abbreviations.

Additional file 11, Table S8.

Table S8 is associated with Figure S3D-F and lists all genes differentially expressed between two clusters (among HB.2, CHB and SC).
